# RalA and RalB relocalization to depolarized mitochondria depends on clathrin-mediated endocytosis and facilitates TBK1 activation

**DOI:** 10.1371/journal.pone.0214764

**Published:** 2019-04-17

**Authors:** Sarah R. Pollock, Austin R. Schinlever, Ali Rohani, Jennifer A. Kashatus, David F. Kashatus

**Affiliations:** Department of Microbiology, Immunology and Cancer Biology, University of Virginia School of Medicine, Charlottesville, Virginia, United States of America; The University of British Columbia Life Sciences Institute, CANADA

## Abstract

Healthy mitochondria use an electrochemical gradient across the inner mitochondrial membrane (IMM) to generate energy in the form of ATP. A variety of endogenous and exogenous factors can lead to transient or sustained depolarization of the IMM, including mitochondrial fission events, expression of uncoupling proteins, electron transport chain (ETC) inhibitors, or chemical uncouplers. This depolarization in turn leads to a variety of physiological responses, ranging from selective mitochondrial clearance (mitophagy) to cell death. How cells recognize and ultimately respond to depolarized mitochondria remains incompletely understood. Here we show that the small GTPases RalA and RalB both relocalize to mitochondria following depolarization in a process dependent on clathrin-mediated endocytosis (CME). Furthermore, both genetic and pharmacologic inhibition of RalA and RalB leads to an increase in the activity of the atypical IκB kinase TBK1 both basally and in response to mitochondrial depolarization. This phenotype was also observed following inhibition of Ral relocalization. Collectively, these data suggest a model in which RalA and RalB inhibit TBK1 and that relocalization of Ral to depolarized mitochondria facilitates TBK1 activation through release of this inhibition.

## Introduction

The Ras-related small GTPases RalA and RalB are directly activated downstream of active Ras. Interest in Ral proteins has grown in recent years as a number of studies have demonstrated aberrant activation of Ral in several human cancer tissues and cell lines [1–4]. Importantly, genetic or pharmacological inhibition of Ral blocks tumor growth in a variety of models [5–7], suggesting Ral may be an attractive therapeutic target for human cancer.

RalA and RalB share 80% sequence identity at the amino acid level and function through the engagement of a shared set of effector proteins. Despite this, RalA and RalB exhibit both redundant and distinct functions, and this specificity may be regulated through distinct post-translational modifications and subcellular localization. Key Ral effectors include RalBP1, a large multifunctional protein that regulates endocytosis and mitotic mitochondria fission [8], among other cellular processes. Other Ral effectors include Sec5 and Exo84, components of the exocyst complex [9,10] that regulate exocytosis in polarized cells, but can also contribute to both cell survival and autophagy [11,12]. Sec5 and Exo84 also link Ral activity to that of Tank-binding kinase 1 (TBK1), a non-canonical IκB kinase that regulates a variety of cellular processes including autophagy and the inflammatory response. Several reports have linked overexpression of a constitutively active RalB mutant to a Sec5-dependent increase in TBK1 activity in the context of viral infection and oncogene activation [13,14]. Moreover, TBK1 phosphorylation of Exo84 leads to dissociation of the RalA/Exo84 complex to promote Glut4 trafficking during insulin signaling [15].

TBK1 is also recruited to mitochondria to promote mitophagy, the selective autophagic removal of damaged mitochondria from the cell [16]. Mitochondria accumulate damage through a variety of both normal and pathological processes. Damaged mitochondria must be removed to maintain cellular health and are associated with a variety of diseases, including neurodegenerative diseases such as Parkinson’s disease and ALS [17,18]. Given that Ral signals to affect autophagy, it is possible that Ral may also be implicated in mitophagy.

Changes in mitochondrial dynamics and mitophagy can be induced by collapse of the proton gradient across the inner mitochondrial membrane [19]. Mitochondrial membrane potential drives ATP synthesis. Consequently, sustained changes in mitochondrial membrane potential indicate damage and result in mitophagy [20]. In contrast, transient mitochondrial depolarization induces changes in mitochondrial dynamics and regulates the timing in which mitochondria are able to enter the fission-fusion cycle. During mitochondrial fission, mitochondria experience transient mitochondrial depolarization [20]. Interestingly, one daughter mitochondria experiences greater transient depolarization than the other and undergoes a recovery period where its mitochondrial membrane potential is restored before it can fuse with other mitochondria [20].

Although mitochondrial depolarization can lead to these changes in mitochondrial dynamics and mitophagy, the molecular response to depolarization is not well understood. Given that changes in mitochondrial dynamics and mitophagy are associated with neurodegenerative diseases and cancers, it is imperative that we elucidate the cellular response to mitochondrial depolarization. Moreover, the fact that Ral is involved in mitochondrial dynamics [8] and may be involved in mitophagy, potentially implicates Ral during mitochondrial depolarization. Therefore, we sought to determine Ral’s role in response to mitochondrial depolarization.

We demonstrate that both RalA and RalB relocalize to depolarized mitochondria. We find that pharmacologic inhibition of clathrin-mediated endocytosis (CME) impairs RalA and RalB localization to depolarized mitochondria, suggesting that CME facilitates Ral trafficking following mitochondrial depolarization. Consistent with this finding, genetic inhibition of CME results in impaired RalA recruitment to depolarized mitochondria. Additionally, we demonstrate that both pharmacological and genetic inhibition of both RalA and RalB leads to increased TBK1 activity, suggesting that Ral negatively regulates TBK1 activity. Collectively, our data support a model where RalA and RalB localize to depolarized mitochondria in a CME-dependent process and regulate TBK1 activity.

## Materials and methods

### Chemicals

Carbonyl cyanidem-3-chlorophenylhydrazone (CCCP) was obtained from Calbiochem. Antimycin A was obtained from Sigma-Aldrich (ref. A8674). Oligomycin was obtained from MP Biomedicals, LLC (ref. 151786). Valinomycin was obtained from Thermo Fisher Scientific (ref. V1644). HeLa cells were treated with 10 μM oligomycin and 4 μM antimycin a, 10 μM valinomycin or 10 μM CCCP. Pitstop 2 was obtained from Abcam and used at 20 μM. BX795 was obtained from Enzo Life Sciences, Inc. (ref. 189–0005) and used at 2 μM. BQU57 was obtained from Selleck Chemicals (ref. S7607) and used at 50 μM. DMSO was obtained from VWR (ref. 0231). 16% formaldehyde was obtained from Cell Signaling Technology (ref. 12606S).

### Cell culture

HeLa cells were obtained from the ATTC. HeLa cells were maintained in Dulbecco’s Modified Eagle Medium (DMEM) (ref. 111965092, Life Technologies) supplemented with 10% Fetal Bovine Serum (FBS, ref. 16000044, GIBCO) and 1% Penicillin-Streptomycin (ref. 15140122, GIBCO). HeLa cells stably expressing mCherry-Parkin were generated using a retroviral system and selected using 2.5 μg/mL Blasticidin (ref. R21001, GIBCO). shScramble, shRalA/RalB and shAP50 HeLa cells were generated using a retroviral system and selected using 1 μg/mL Puromycin (ref. p8833, Sigma-Aldrich), while shRalA/RalB cells were further selected with 500 μg/mL Neomycin (ref. 10131035, GIBCO). shRalA/RalB stably expressing WT Flag RalA (shRNA resistant) were selected with 180 μg/mL Hygromycin B (ref. 10687010, Thermo Fisher Scientific). Cells were tested for mycoplasma with the MycoAlert PLUS Mycoplasma Detection Kit (ref. LT07-701, Lonza).

### Transfection/Immunocytochemistry

30,000 HeLa cells were seeded onto glass microslides (#1.5) (ref. 12541B, Fisher Scientific) and transfected 2 days later with the indicated constructs using FuGENE 6 (ref. E269A, Promega). 24 hours post-transfection cells were treated as described with either CCCP or OA and fixed with 4% formaldehyde/PBS for 5 minutes at room temperature. Cells were mounted either with Prolong Gold Antifade Mountant with DAPI (ref. P36931, Invitrogen) or Prolong Diamond Antifade Mountant (ref. P36930, Invitrogen).

### RNA interference and constructs

Oligonucleotides designed to generate short hairpin RNAs (shRNA) targeting the μ2 subunit of AP2 (aka AP50) (5’-GTGGATGCCTTTCGGGTCA-3’)[21] were cloned into pSUPER.retro.puro (OligoEngine, Inc.). pSUPER.retro.puro.RalA (shRalA—1: 5’- AAGACAGGTTTCTGTAGAAGA-3’, and pSUPER.retro.puro.RalB (5’- GACTATGAACCTACCAAAG-3’) were described previously [7]. S194A Flag RalA pbabe puro was made shRNA resistant using the following primers: 5’- TTTAGAAGATAAAAGGCAAGTCAGCGTGGAGGAGGCAAAAAACAGA-3’ and 5’- TCTGTTTTTTGCCTCCTCCACGCTGACTTGCCTTTTATCTTCTAAA-3’. This construct was then cloned into pbabe hygro by cutting pbabe hygro with BAMHI and SalI, while the insert was cut with BamH1 and XhoI. Upon obtaining S194A Flag RalA pbabe hygro, site-directed mutagenesis was performed to convert S194A Flag RalA to wild-type RalA using the following primers: forward- 5’-AAAAAGAAGAGGAAAAGTTTAGCCAAGAGAAT-3’ and reverse- 5’-ATTCTCTTGGCTAAACTTTTCCTCTTCTTTTT-3’. pSR neo RalB was a gift from Chris Counter (shRalB: 5’GACTATGAACCTACCAAAG -3’). HeLa cells were transiently transfected with FuGENE 6 (ref. E269A, Promega) using 500 ng or 250 ng of the indicated fluorescent constructs: pEGFP (Clonetech Laboratories, Inc.), pEGFP-C1-RalA, pEGFP-C1-RalB, mCherry-Parkin, mito-YFP or mito-BFP. mCherry-Parkin was a gift from Richard Youle (Addgene plasmid # 23956). mCherry-Parkin was cloned into pWZL-blasti using the In-Fusion HD Cloning Kit (Clontech Laboratories, Inc.). mito-BFP was a gift from Gia Voeltz (Addgene plasmid # 49151). mito-BFP was cloned into pBABE-puro (Millenium Pharmaceuticals) using XhoI and EcoR1. A Kozak sequence and EcoR1 site was introduced into mito-BFP using site-directed mutagenesis (Forward primer: 5’ TGCAGTCTCGAGGCCGCCACCATGCTTTCACTACGTCAA-3’ and Reverse primer: 5’-GCTGACGAATTCTCAATTAAGCTTGTGCCC-3’). All stable cell lines were generated using the retroviral packaging vector, pCL-10A1 from Imgenex. pGEX-KG-RalBD was used to generate expression of glutathione *S*-transferase (GST)-RalBD[22]. S183A, S194A, S194A/S183A, S28N and Q72L mutant GFP-RalA constructs were generated from performing site-directed mutagenesis.

### Western blotting

Whole cell lysates were prepared in RIPA buffer with protease inhibitors (2 μg/mL Aprotinin, 2 μg/mL Leupeptin, 1 mM PMSF, 1 mM Na_3_VO_4_ and 50 mM NaF) and lysates were quantified using Bio-Rad Protein Assay Dye Reagent Concentrate (ref. 5000006).

Equivalent protein amounts (generally 30 or 50 μg) were resolved by freshly cast 10% SDS-PAGE gels. Gels were run with either EZ-Run Prestained *Rec* Protein Ladder (ref. BP3603500, Fisher Scientific) or SeeBlue Plus2 Pre-stained Protein Standard (ref. LC5925, Invitrogen). Gels were transferred to PVDF membranes (ref. IPVH00010, Immobilin-P, Millipore) and immunoblotted with the following antibodies: mouse monoclonal α-AP50 (ref. 611350, BD Transduction Laboratories, 1:500), mouse monoclonal α-RalA (ref. 610221, BD Transduction Laboratories, 1:1000), rabbit monoclonal α-GAPDH (ref. 5174, Cell Signaling Technology, 1:5000), mouse monoclonal α-OPA1 (ref. 612606, BD Transduction Laboratories, 1:1000), rabbit monoclonal α-β-Actin (ref. 4970, Cell Signaling Technology, 1:2000), rabbit monoclonal α-phospho-TBK1 (Ser172) (ref. 5483, Cell Signaling Technology, 1:1000), rabbit monoclonal α-β-tubulin (ref. 2128, Cell Signaling Technology, 1:2000) and mouse monoclonal α-RalB (ref. 04–037, Millipore Sigma, 1:500). Goat α-rabbit- or goat α-mouse-HRP secondary antibodies were used (ref. 111-036-046, ref. 115-036-008, Jackson ImmunoResearch Laboratories, Inc., 1:5000) for visualization of blots enhanced chemiluminescence detection (WesternBright ECL). Band pixel intensity was quantified by using ImageJ and bands were normalized to the indicated controls.

### Pitstop 2 experiments

50,000 HeLa cells were plated onto glass microslides (#1.5) (ref. 12541B, Fisher Scientific). Two days later, cells were transfected with 500 ng each of the indicated constructs using a 3:1 ratio of FuGENE 6 (ref. E269A, Promega) to DNA. 24 hours post-transfection, cells were washed twice with 1X PBS and pre-treated with either DMSO or 20 μM Pitstop 2 (ref. ab120687) in serum-free DMEM supplemented with 10 mM HEPES (ref. 15630080, GIBCO) for 15 minutes at 37°C. Following the 15 minute pre-treatment, cells were treated with one of the following ways for 1 hour at 37°C: 1. DMSO, 2. 10 μM oligomycin and 4 μM antimycin a, 3. 20 μM Pitstop 2 or 4. 20 μM Pitstop 2 and 10 μM oligomycin and 4 μM antimycin a. Cells were then washed twice with 1X PBS and fixed with 4% formaldehyde/PBS for 5 minutes at room temperature. For image analysis, over 50 cells were counted per treatment to examine Parkin colocalization with the mitochondria (n = 3).

### Transferrin-uptake

#### Microscopy

50,000 HeLa cells were plated onto glass microslides (#1.5) (ref. 12541B, Fisher Scientific). Two days later, cells were washed twice with 1X PBS and treated for 15 minutes at 37°C with 0.1% DMSO or 20 μM Pitstop 2 in serum-free DMEM supplemented with 10 mM HEPES. Following the 15 minute pre-treatment, cells were treated with 10 μg/mL Transferrin Alexa Fluor-647 (ref. T23366, Life Technologies) in the presence or absence of 20 μM Pitstop 2 in serum-free DMEM supplemented with 10 mM HEPES for 15 minutes at 37°C. Cells were washed twice with 1X PBS and fixed with 4% formaldehyde/PBS for 5 minutes at room temperature.

#### Flow cytometry

For flow cytometric analysis of transferrin uptake, shScramble and shAP50 HeLa cells were serum-starved for 30 minutes in serum-free DMEM containing 25 mM HEPES and 0.5% Bovine Serum Albumin (BSA) (ref. BSA-50, Rockland Immunochemicals Inc.). Cells were detached using 10 mM EDTA/PBS, collected in serum-free DMEM and spun for 3 minutes at 1000 rpm at 4°C. Cells were then resuspended in cold serum-free DMEM containing 25 mM HEPES, 0.5% BSA and 50 μg/mL Transferrin Alexa Fluor-647. Cells were incubated at 4°C for 10 minutes and moved to 37°C for 15 minutes. Cells were then spun for 3 minutes at 1000 rpm at 4°C and washed twice with 1mL of cold 1X PBS/0.5% BSA. Following cold PBS/0.5% BSA washes, cells were acid washed twice with cold acidic buffer (0.1M glycine, 150 mM NaCl, pH 3). Cells were then washed with cold 1X PBS and fixed in 2% formaldehyde/PBS for 10 minutes at room temperature. Following fixation, cells were washed with cold 1X PBS, spun and resuspended in 500 μl cold 1X PBS. Unstained cells were also prepared as a negative control for flow cytometric analysis.

### Confocal microscopy

Fixed cells were mounted onto glass microslides (#1.5) (ref. 12541B, Fisher Scientific) and imaged on a Zeiss LSM 710 confocal microscope using the 63X oil objective. Single color controls were tested to asses bleedover. For quantification of RalA or RalB recruitment to mitochondria in HeLa cells, 25 random images across 3 trials were analyzed. To quantify the colocalization of Ral and the mitochondria, we separated Ral and mitochondria from their respective background signals. Given that mitochondria and Ral contain exhibit different staining patterns, we separated different channels and saved each channel as an independent image. Following this, multiple preprocessing steps, including median, and top-hat filtering were applied to remove noise and other imperfections in each image. After preprocessing, a simple thresholding method was used for segmentation of the mitochondrial network and the RalA or RalB protein distribution. Threshold levels were chosen that effectively removed the noisy background from the real objects. The result of the thresholding step is a binary mask, with the value of 0 for background, and a value of 1 for the foreground mitochondrial or RalA or RalB contents.

Using the thresholded images, we determined percentage of overlap between the mitochondrial network and RalA or RalB, which we termed percent overlap. This was calculated using the following formula:

$$Overlap\;=\;\frac{common}{union}$$

The numerator is equal to the total number of pixels with the value of 1 after applying a logical AND between two images. These are the pixels where both mitochondria and RalA or RalB have a value of 1 after thresholding. The denominator is the sum of pixel values after applying a logical OR between the thresholded channels. The pixels where either the mitochondria or RalA or RalB exist will have the value of 1 after applying the OR operation. Using this formula, the overlap has a value between 0 and 1, where 0 means no overlap, while the value of 1 means a complete overlap. By multiplying this value by 100, the overlap is represented as a percentage.

The other feature defined and used to quantify the colocalization of the mitochondria and RalA or RalB is the proximity index, which has a value between 0 and 1. The proximity index represents the similarity in the spatial distribution of the mitochondrial network and Ral proteins. The proximity index of 1 represents two completely overlapping structures, while an index of zero represents no similarity or absence of one channel.

To calculate the proximity index, we first average the values of the thresholded channels at each pixel. As a result, the pixels where both mitochondria and RalA or RalB exist (overlap) will have a value of 1, the pixels where either mitochondria or RalA or RalB exist will have the value of 0.5, and finally, the pixels where none of them exist, the pixel value will be 0. Following the averaging, we define a 2^*k*^ × 2^*k*^, 2 ≤ *k* ≤10, sliding window to sweep the entire averaged image from top left to bottom right.

Starting from window one, we calculate the ratio of mitochondria to RalA or RalB and vice versa. The minimum of these ratios with a value between 0 and 1 is selected as the ratio factor for that window. Following this step, the ratio factor is multiplied to each individual pixel in the window. Finally, the average of the non-zero, weighted pixels in the window is defined as the proximity index for that window and is stored for subsequent measurements. This process is repeated for each window until the whole image is scanned by the sliding window. Ultimately, the proximity index of RalA or RalB and mitochondria in the image is determined by summing up the proximity indices from different windows and dividing the result by the number of the windows with a non-zero proximity index.

The overlap measurement is independent of window size, but the proximity index is a function of the windows size as described earlier. Different window sizes define the proximity index at different granularities. By calculating and averaging the proximity indices of multiple cells with different treatments at different windows size we can identify the window size that results in the widest distinction between the treatment groups and use it to compare different treatments. We used a window size of 64 for our analyses.

### RalA-GTP pulldown

GST-RalBD beads were generated as follows: 300 mL of BL21 bacteria expressing pGEX-KG-RalBD were induced with IPTG and rocked for 3 hours at 37°C. Bacteria were then spun at 4°C at 6,000 g for 15 minutes and lysed with 40 mL of PBST with inhibitors (1% aprotinin, 1 mM PMSF and 0.5 mM DTT). Bacteria were sonicated (Output 4, Constant 50, 30 hits for 3 times) and spun at 4°C at 12,000 rpm for 20 minutes. Supernatant was combined with 500 μl of Glutathione Sepharose 4B (ref. 17-0756-01, GE Healthcare) beads and rocked overnight at 4°C. The beads were then spun at 2,000 rpm for 2 minutes each and washed 3 times with 10 mL of lysis buffer.

To perform the GST-RalBD pulldown, cells were serum starved overnight and treated with 10 μM CCCP for 0, 30, 60 and 80 minutes. Cells were washed two times with cold 1X PBS, scraped and spun at 1,000 rpm for 5 minutes. Cells were lysed using 200 μl of GTP lysis buffer (1% NP-40, 50 mM Tris pH 7.5, 200 mM NaCl, 10 mM MgCl_2_, 1% aprotinin, 1 mM PMSF, 0.5 mM DTT) on ice for 30 minutes. Samples were spun at 14,000 rpm for 10 minutes and a BCA was performed to quantify protein amounts. 500 μg and 100 μg of protein were used for pulldowns and inputs, respectively. For GST-RalBD pulldowns, 500 μg of protein was combined with 30 μl of GST-RalBD in a total volume of 500 μl of GTP lysis buffer, rotating at 4°C for 45 minutes. Samples were washed two times with 500 μl of GTP lysis buffer and spun at 2,000 rpm for 2 minutes. Total RalA and GTP-RalA levels were detected by SDS-PAGE and immunoblotting.

## Results

### RalA and RalB relocalize to mitochondria in response to mitochondrial depolarization

**Fig 1:**
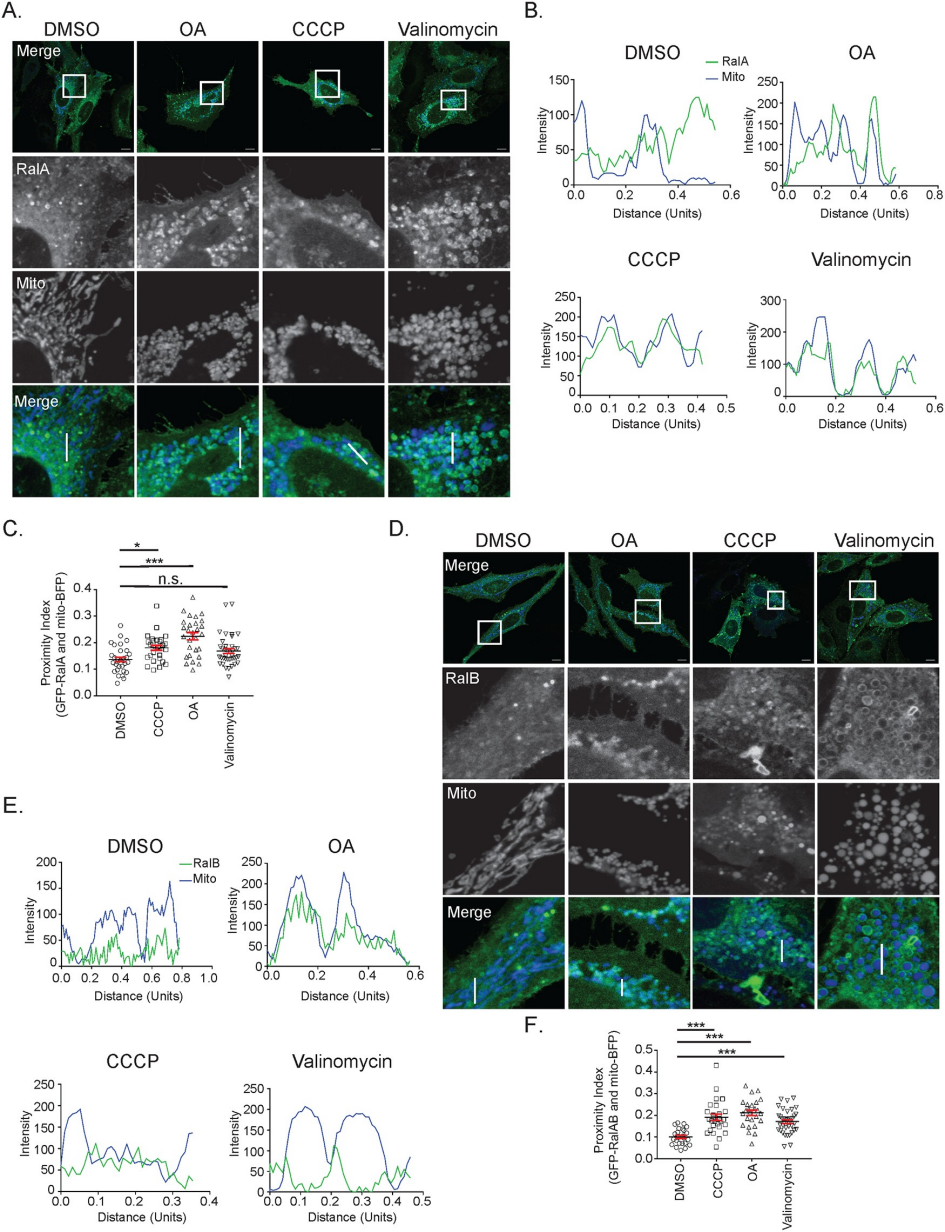
Both RalA and RalB are recruited to depolarized mitochondria. (A) HeLa cells transiently expressing mito-BFP and GFP-RalA were treated with vehicle control (DMSO), 10 μM oligomycin and 4 μM antimycin a (OA), 10 μM CCCP or 10 μM valinomycin for 3 hours and imaged with confocal microscopy (Scale bars = 10 μM). (B) Plot profiles of cells in A (1 Unit = 96 pixels). White lines in A indicate sections analyzed using ImageJ. (C) Quantification of RalA distance to mitochondria (One-way ANOVA using Dunnett’s multiple comparisons test, mean ± SEM, *p ≤ 0.05, ***p ≤ 0.0001, n.s. = not significant, n = 3). (D) HeLa cells transiently expressing mito-BFP and GFP-RalB were treated as in A. (E) Plot profiles of cells in D (1 Unit = 96 pixels). White lines in D indicate sections analyzed using ImageJ. (F) Quantification of RalB distance to mitochondria (One-way ANOVA using Dunnett’s multiple comparisons test, mean ± SEM, ***p ≤ 0.0001, n = 3).

To identify if RalA is involved in the cellular response to mitochondrial damage, we examined RalA localization in HeLa cells that transiently express mito-BFP and GFP-RalA. Mitochondrial depolarization was induced for 3 hours using 3 different chemical means: 1. oligomycin (ATP synthase inhibitor) and antimycin a (inhibits complex III of the electron transport chain) (OA), 2. CCCP (protonophore) and 3. valinomycin (potassium-specific ionophore). In untreated cells, RalA localizes to both internal structures and the plasma membrane (Fig 1A). Following treatment with OA, CCCP and valinomycin, RalA relocalizes to mitochondria or punctate structures adjacent to mitochondria, which become fragmented and accumulate in a perinuclear fashion (Fig 1A). This RalA relocalization is consistent with the three distinct methods of depolarization, though we detect some differences in the nature of the colocalization that may reflect different effects the drugs have on mitochondrial structure. For example, cells treated with OA and valinomycin mainly exhibit distinct ring-like structures of RalA around the mitochondria (Fig 1B). In contrast, cells treated with CCCP tend to display perinuclear RalA puncta adjacent to mitochondria (Fig 1B).

Instances where RalA is directly colocalized with mitochondria in response to OA treatment can be quantified by calculating the percent overlap of RalA with mitochondria. However, the percent overlap calculation is not able to capture instances where RalA is in the proximity of mitochondria when cells are treated with valinomycin or CCCP. Therefore, we quantified the spatial relationship of RalA to mitochondria using a proximity index score to measure the distance of RalA to mitochondria (see Materials and methods). We observe a statistically significant increase in RalA proximity to mitochondria following CCCP and OA treatment (Fig 1C). We also observe an increase in RalA proximity to mitochondria following valinomycin treatment, although this was not statistically significant (p-value = 0.06) (Fig 1C).

We next examined if the RalA homologue RalB also relocalizes to depolarized mitochondria by examining HeLa cells that transiently express mito-BFP and GFP-RalB. Prior to mitochondrial depolarization, RalB localizes primarily to membrane-bound compartments within the cytoplasm, similar to RalA, with some RalB at mitochondria (Fig 1D and 1E). Interestingly, like RalA, RalB relocalizes to mitochondria following 3 hours of treatment with OA, CCCP or valinomycin. Once again, we observe differences in the appearance of RalB localization to mitochondria depending on the depolarization method. Cells treated with OA and valinomycin largely exhibit ring-like structures of RalB around mitochondria with some direct colocalization (Fig 1D and 1E). Cells treated with CCCP mainly display RalB puncta adjacent to mitochondria (Fig 1D and 1E), which is similar to RalA localization in response to CCCP treatment. We also observe statistically significant increases in RalB proximity to mitochondria following OA, CCCP and valinomycin treatment (Fig 1F).

To confirm that OA, CCCP and valinomcyin induce mitochondrial depolarization, we repeated the above experiments in HeLa cells that stably or transiently express mCherry-Parkin and transiently express mito-BFP and/or GFP-RalA. mCherry-Parkin was used as a positive control for mitochondrial depolarization as Parkin translocates from the cytosol to mitochondria in response to mitochondrial depolarization [23]. In response to OA, CCCP and valinomcyin, Parkin is recruited to the mitochondria indicating that mitochondrial depolarization is induced (S1A, S1B and S1C Fig). Moreover, RalA relocalization to depolarized mitochondria occurs in a similar fashion to cells that lack exogenous Parkin, indicating that Parkin expression does not affect RalA redistribution (S1A, S1B and S1C Fig). Importantly, GFP alone remains cytosolic before and after OA treatment, indicating that the signal promoting GFP-RalA mitochondrial redistribution is within the Ral sequence (S2A Fig). Collectively, these data indicate that both RalA and RalB relocalize to depolarized mitochondria.

### RalA-GTP levels decrease following OA and CCCP treatment

**Fig 2:**
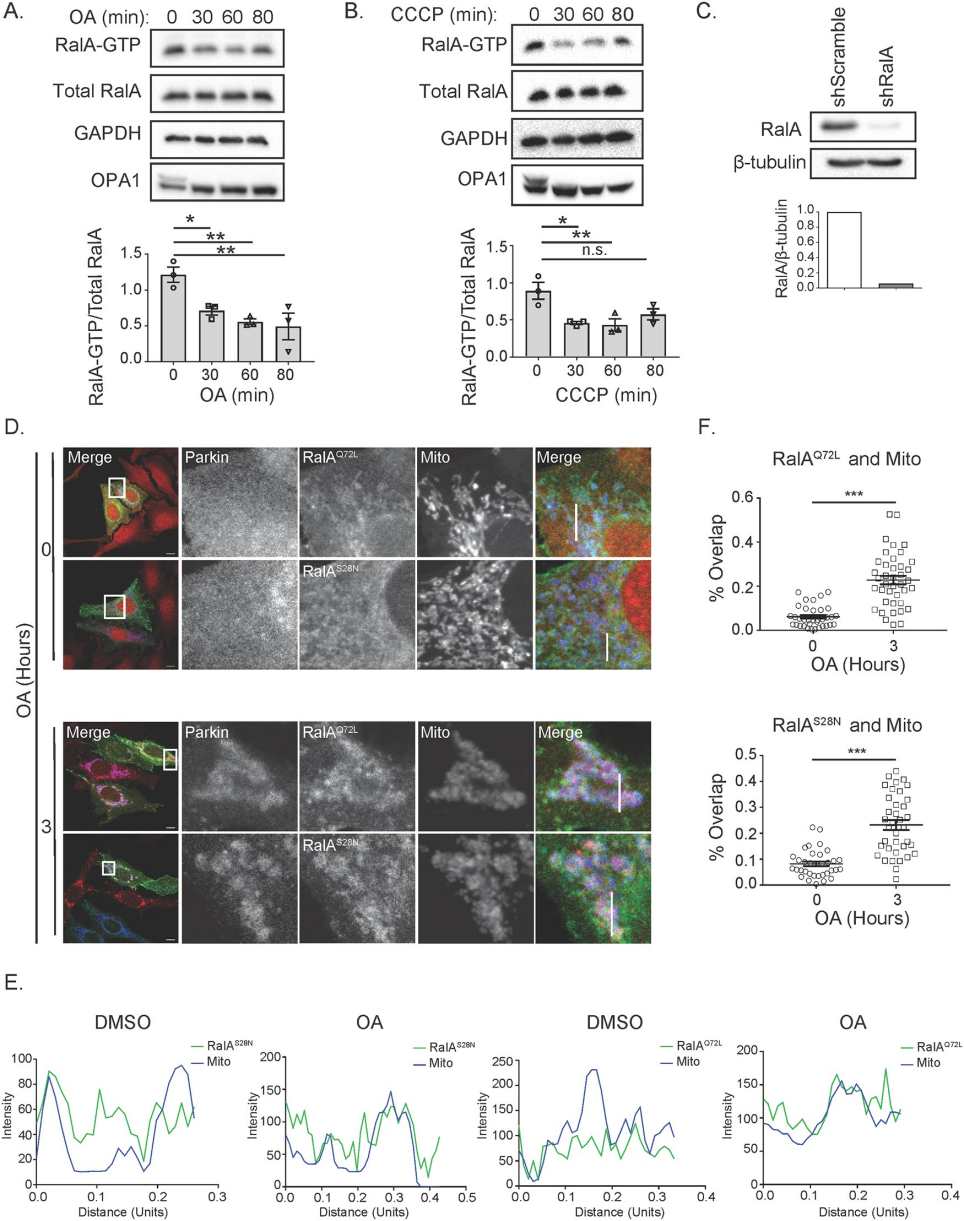
RalA GTP-loading decreases following mitochondrial depolarization, but does not regulate RalA localization. (A) HeLa cells were treated over an 80-minute time course with 10 μM oligomycin and 4 μM antimycin a (OA) and Ral-GTP levels were assessed by GST-RalBD pulldown. Ral-GTP levels were quantified and normalized to total RalA (One-way ANOVA using Dunnett’s multiple comparisons test, mean ± SEM, *p ≤ 0.05, ** p ≤ 0.005, n = 3). (B) Experiment in A was repeated using 10 μM CCCP (One-way ANOVA using Dunnett’s multiple comparisons test, mean ± SEM, *p ≤ 0.05, **p ≤ 0.01, n = 3). (C) Immunoblot analysis of RalA in HeLa cells stably expressing the indicated shRNA constructs. RalA levels were quantified and normalized to β-tubulin. (D) HeLa cells stably expressing mCherry-Parkin and transiently expressing mito-BFP and the indicated RalA-GTP mutants were treated with OA over 3 hours and imaged by confocal microscopy (Scale bars = 10 μM). (E) Plot profiles of cells in D (1 Unit = 96 pixels). White lines in D indicate sections analyzed using ImageJ. (F) Quantification of RalA and mitochondria colocalization in D (Two-tailed Unpaired t-test, mean ± SEM, ***p ≤ 0.0001, n = 3).

To further examine RalA activity in response to mitochondrial damage, we next sought to determine how depolarization affects RalA-GTP loading. RalA-GTP levels significantly decrease over 80 minutes of OA treatment (Fig 2A). We used OPA1 as a readout to confirm mitochondrial depolarization since OPA1 is preferentially processed into its shorter isoform following depolarization (Fig 2A and 2B) [24]. We observed similar decreases in RalA-GTP levels following CCCP treatment (Fig 2B). Changes in RalA-GTP levels may be a direct consequence of the loss in mitochondrial activity, as decreased ATP synthesis should result in decreased cellular GTP levels. Alternatively, RalA relocalization may alter its proximity to its guanine nucleotide exchange factors (GEFs) or GTPase activating proteins (GAPs). To determine if RalA-GTP loading affects its relocalization following mitochondrial damage, we co-transfected shRalA HeLa cells (Fig 2C) that stably express mCherry-Parkin with mito-BFP and shRNA resistant GFP-RalA^Q72L^ (constitutively GTP-bound) or GFP-RalA^S28N^ (constitutively GDP-bound) [25–27]. Following OA treatment, both GFP-RalA^S28N^ and GFP-RalA^Q72L^ mutants relocalize with Parkin to the mitochondria (Fig 2D, 2E and 2F). These data indicate that nucleotide-binding neither positively nor negatively regulates RalA localization following mitochondrial depolarization. Further, the data suggest that direct effectors of Ral, which bind to the GTP-bound form, are not required for Ral relocalization.

### Phosphorylation of neither serine 194 nor serine 183 is required for RalA relocalization following mitochondrial depolarization

Phosphorylation of the C-terminal hypervariable domain of RalA has been shown to regulate its localization, potentially due to the negatively charged phosphate group neutralizing the charge of the polybasic region [8,28]. Neutralizing the charge changes RalA’s affinity for membranes, similar to what has been demonstrated for KRas [29]. RalA is phosphorylated on serine 194 and serine 183 by Aurora A and potentially PKC, respectively [30,31]. PP2A Aβ dephosphorylates RalA on serine 194 and serine 183 [32].

**Fig 3:**
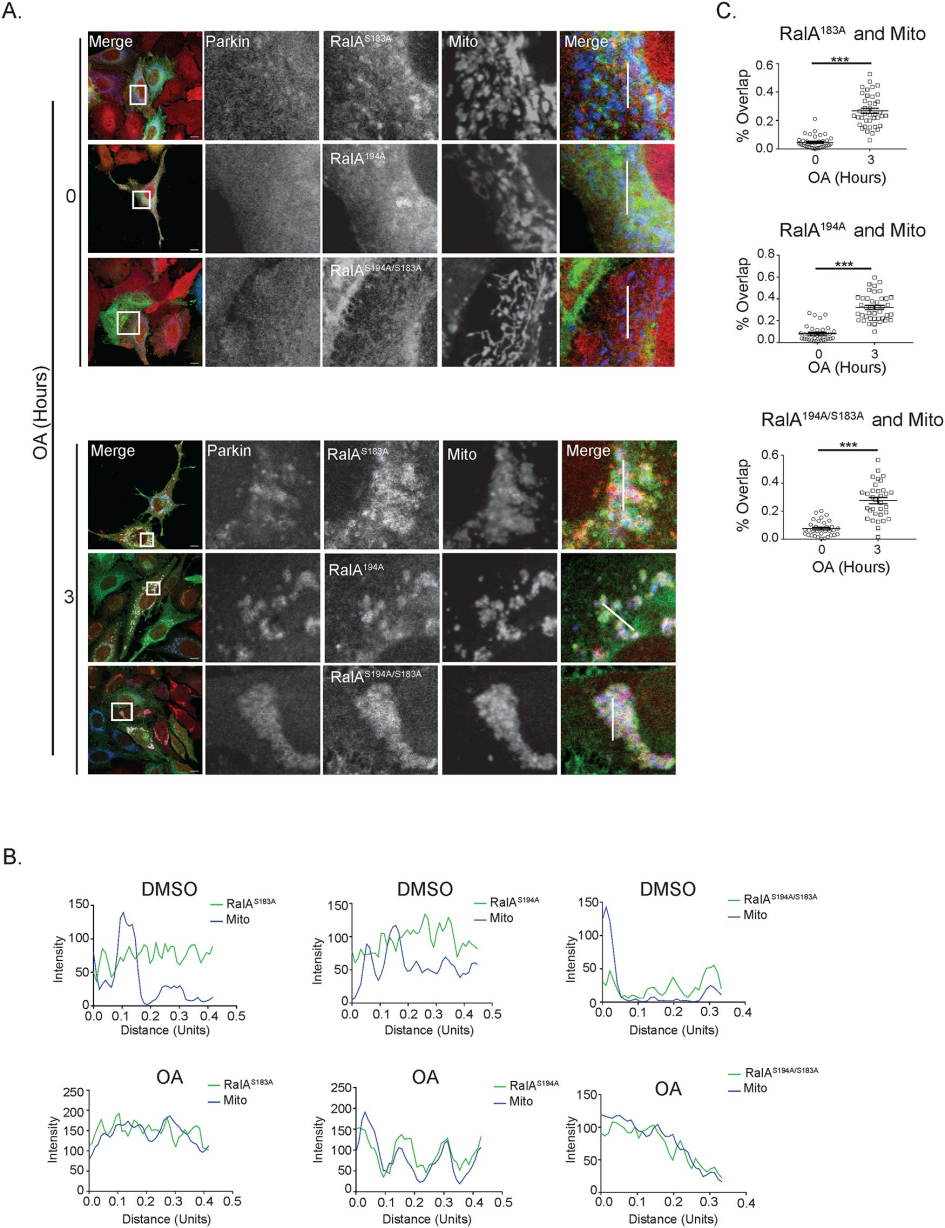
Phosphorylation of RalA does not regulate RalA subcellular localization in response to mitochondrial damage. (A) HeLa cells that stably express mCherry-Parkin and transiently express mito-BFP and the indicated GFP-RalA phosphorylation mutants were treated with 10 μM oligomycin and 4 μM antimycin a for 3 hours and imaged by confocal microscopy. DMSO was used as a vehicle control (Scale bars = 10 μM). (B) Plot profiles of cells in A. White lines in A indicate sections analyzed using ImageJ (1 Unit = 96 pixels). (C) Quantification of RalA and mitochondria colocalization in A (Two-tailed Unpaired t-test, mean ± SEM, ***p ≤ 0.0001, n = 3).

To identify if phosphorylation of RalA regulates RalA relocalization in response to mitochondrial depolarization, we co-transfected mito-BFP and shRNA resistant S183A, S194A or S194A/S183A mutants of GFP-RalA into shRalA HeLa cells stably expressing mCherry-Parkin and treated with OA for 3 hours. Prior to mitochondrial depolarization, we observe S183A, S194A and S194A/S183A mutants of GFP-RalA localize to distinct structures within the cell with some RalA localized to mitochondria (Fig 3A and 3B). However, after 3 hours of OA treatment, all phospho-RalA mutants exhibit a more pronounced perinuclear punctate phenotype and relocalize with Parkin to mitochondria (Fig 3A and 3B). Moreover, all three phospho-RalA mutants exhibit statistically significant colocalization with depolarized mitochondria (Fig 3C). This suggests that phosphorylation of serine 194 and serine 183 are not required for RalA mitochondrial localization following mitochondrial depolarization.

### Clathrin-mediated endocytosis facilitates Ral relocalization to depolarized mitochondria

**Fig 4:**
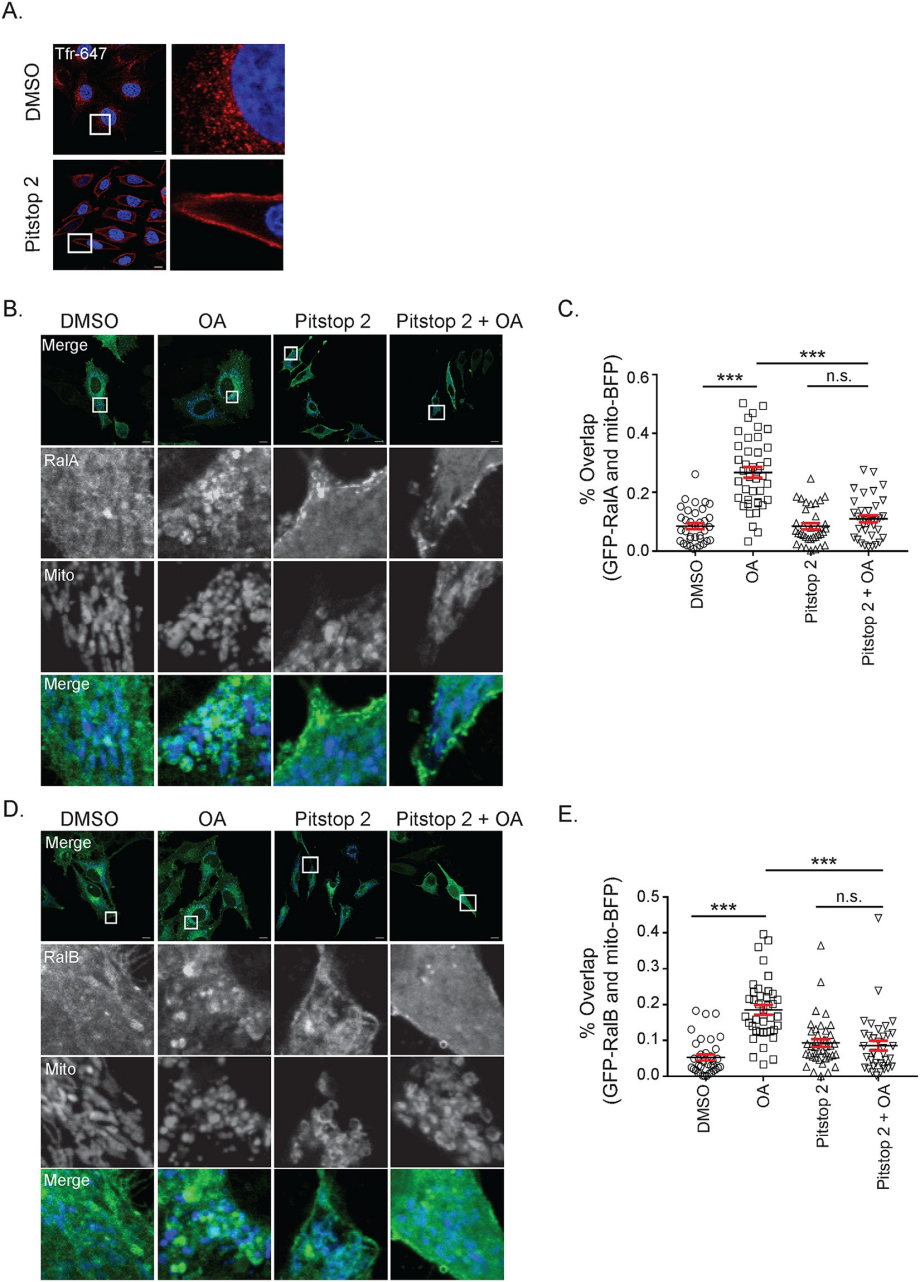
Clathrin-mediated endocytosis facilitates RalA and RalB recruitment to depolarized mitochondria. (A) HeLa cells were treated with 20 μM Pitstop 2 or DMSO (vehicle control) for 15 minutes and transferrin Alexa Fluor-647 uptake was analyzed by confocal microscopy (Scale bars = 10 μM). (B) HeLa cells transiently expressing mito-BFP and GFP-RalA were pre-treated for 15 minutes either with DMSO or 20 μM Pitstop 2 for 15 minutes and then treated for 1 hour with Pitstop 2 and/or 10 μM oligomycin and 4 μM antimycin a (OA) as indicated and imaged by confocal microscopy (Scale bars = 10 μM). (C) Quantification of B to determine RalA colocalization with the mitochondria (One-way ANOVA using Tukey’s multiple comparisons test, mean ± SEM, ***p ≤ 0.001, n.s. = not significant, n = 3). (D) Same as A, except HeLa cells transiently express GFP-RalB instead of GFP-RalA. (E) Quantification of D to determine the degree of RalB colocalization with the mitochondria (One-way ANOVA using Tukey’s multiple comparisons test, mean ± SEM, ***p ≤ 0.001, n.s. = not significant, n = 3).

Ral relocalization has previously been shown to be induced by specific post-translational modifications [33], but as a membrane-anchored protein, it can also relocalize throughout the cell by trafficking through the endo-lysosomal system [34]. As such, we next sought to determine whether endocytic trafficking is required for depolarization-induced RalA relocalization. RalA has previously been identified at sites of clathrin-mediated endocytosis (CME) and its binding partners RalBP1 and PLD are both involved in endocytosis [35–37]. Furthermore, a growing number of studies have demonstrated delivery of specific proteins to mitochondria via endocytic trafficking, including Drp1 and XIAP [38,39]. Thus, to determine whether CME plays a role in the recruitment of Ral to mitochondria following mitochondrial depolarization, we treated HeLa cells transiently expressing mito-BFP and GFP-RalA or GFP-RalB with OA and Pitstop 2, a cell-permeable CME inhibitor, either alone or in combination. Importantly, Pitstop 2 abolishes uptake of fluorescently tagged transferrin, indicating that it effectively inhibits CME at the dose we are using (Fig 4A).

While untreated cells and those treated with Pitstop 2 alone do not exhibit striking RalA and RalB localization at the mitochondria, we observe robust redistribution of RalA and RalB to mitochondria following 1 hour of OA treatment (Fig 4B, 4C, 4D and 4E). Interestingly, cells treated with both OA and Pitstop 2 fail to exhibit Ral localization at mitochondria and in fact, Ral localization in Pitstop 2 treated cells appears shifted towards the plasma membrane (Fig 4B, 4C, 4D and 4E). This data suggests that CME facilitates both RalA and RalB recruitment to depolarized mitochondria.

**Fig 5:**
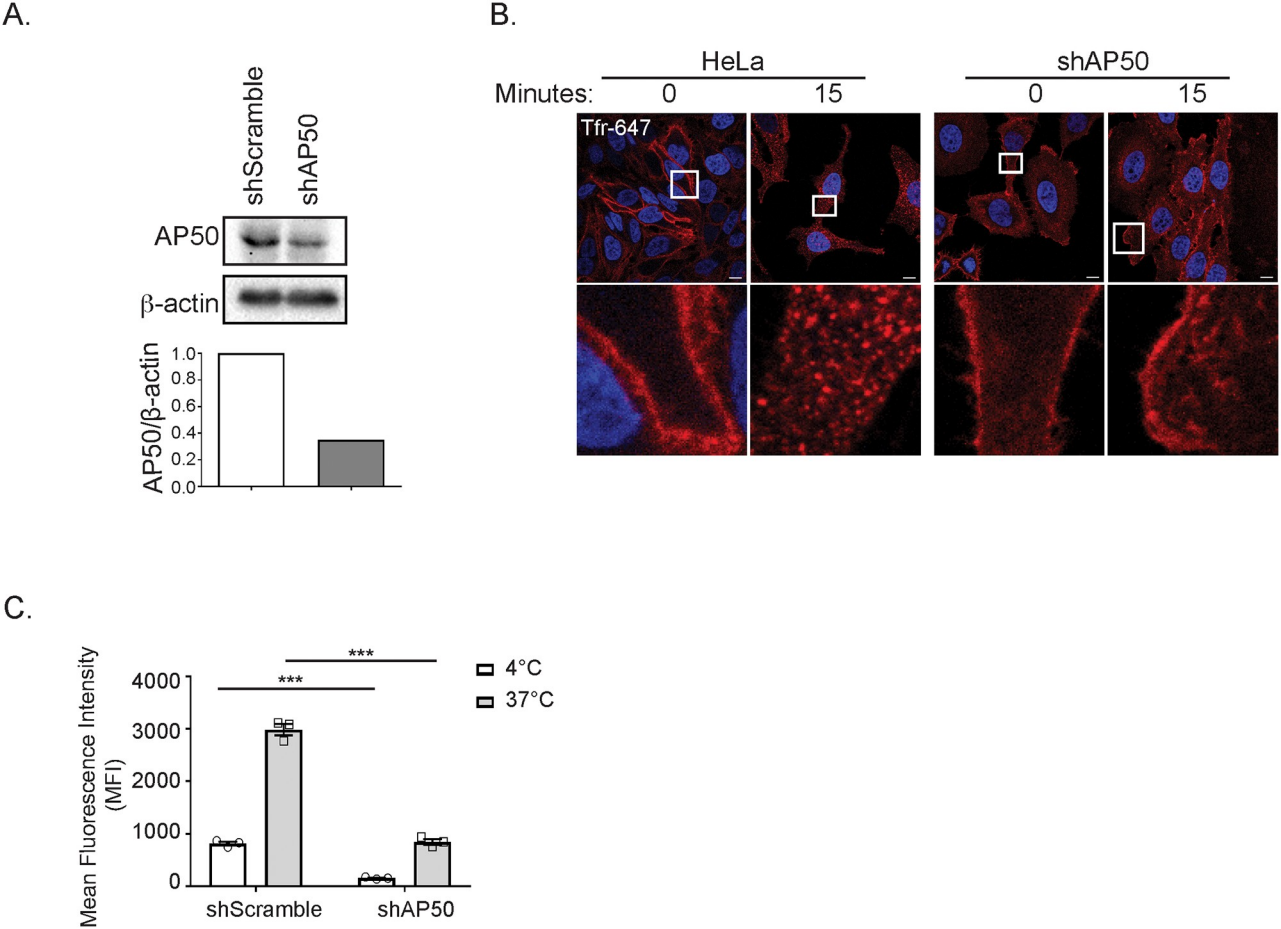
shAP50 cells exhibit impaired clathrin-mediated endocytosis. (A) Immunoblot analysis of AP50 in the indicated cells. AP50 levels were quantified and normalized to β-actin. (B) HeLa cells expressing the indicated shRNA constructs were incubated with transferrin Alexa Fluor-647 and either kept on ice (t = 0) or shifted from 4°C to 37°C for 15 minutes (t = 15) then analyzed by confocal microscopy (Scale bars = 10 μM). (C) Flow cytometric quantification of transferrin uptake in cells treated identically to those in B (Two-way ANOVA using Sidak’s multiple comparisons test, mean ± SEM, ***p ≤ 0.0001, n = 3).

**Fig 6:**
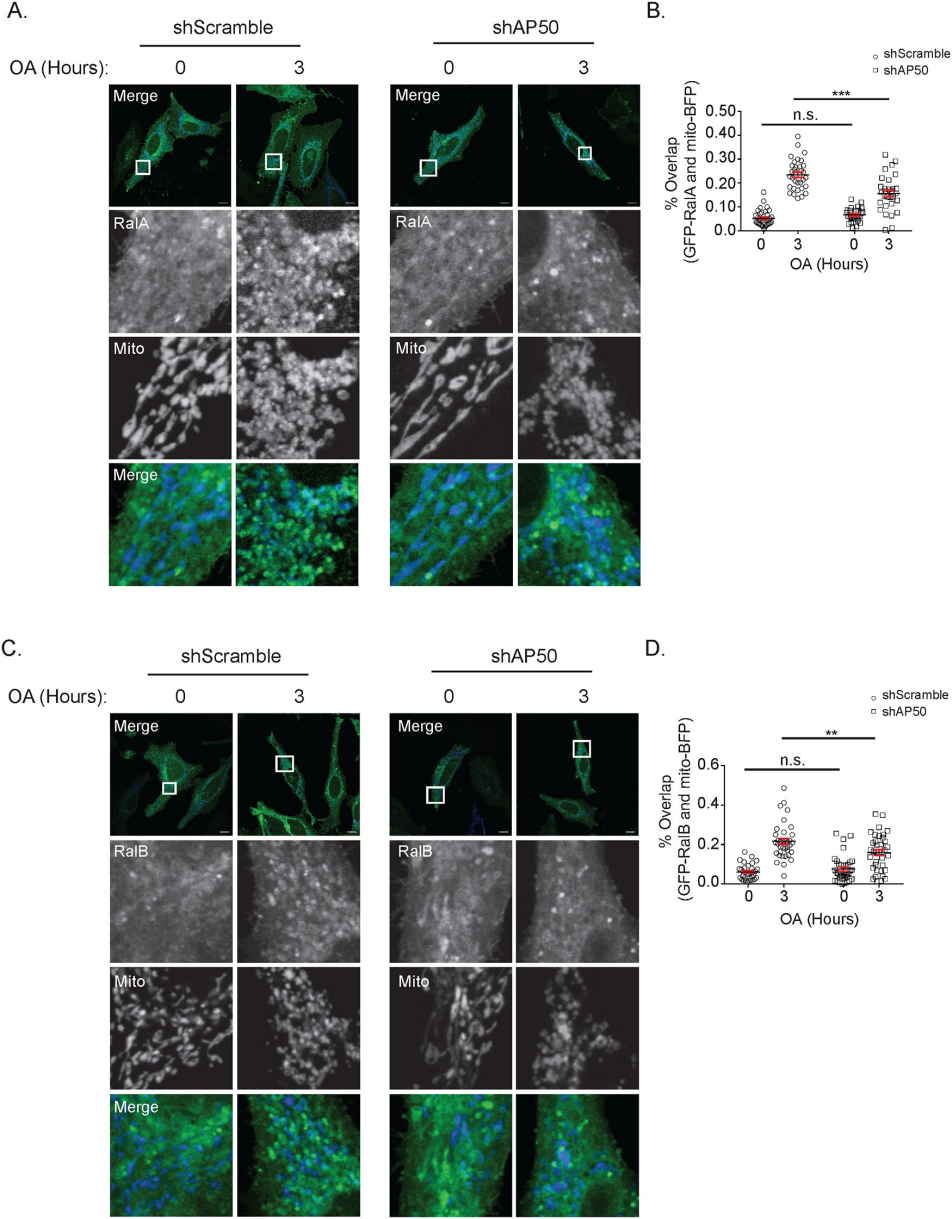
shAP50 cells exhibit impaired RalA and RalB localization to depolarized mitochondria. (A) shScramble and shAP50 HeLa cells transiently expressing GFP-RalA and mito-BFP were treated with 10 μM oligomycin and 4 μM antimycin a (OA) for 3 hours and imaged by confocal microscopy (Scale bars = 10 μM). (B) Quantification of RalA and mitochondria colocalization in A (Two-tailed Unpaired t-test, mean ± SEM, n.s. = not significant, ***p ≤ 0.0001, n = 3). (C) Same as A, except HeLa cells express GFP-RalB instead of GFP-RalA (Scale bars = 10 μM). (D) Quantification of RalB and mitochondria colocalization in C (Two-tailed Unpaired t-test, mean ± SEM, n.s. = not significant, **p ≤ 0.001, n = 3).

Given that clathrin has additional roles outside of CME, and that any pharmacologic inhibitor will exhibit some degree of off-target effects, we examined RalA relocalization in HeLa cells that stably expressed an shRNA targeting AP50, the μ2 subunit of AP2 that is required for clathrin recruitment to sites of endocytosis. We confirmed that shAP50 cells exhibit impaired CME through analysis of transferrin uptake (Fig 5A, 5B and 5C). As expected, both untreated shScramble and shAP50 cells exhibit RalA and RalB localized to structures present throughout the cytoplasm (Fig 6A and 6C). However, following 3 hours of OA treatment, shAP50 cells exhibit significantly impaired RalA and RalB relocalization to depolarized mitochondria compared with shScramble control cells (Fig 6A, 6B, 6C and 6D). Collectively, these findings are consistent with the observed loss of Ral recruitment in cells treated with both OA and Pitstop 2. This supports the notion that CME plays a role in RalA and RalB localization to mitochondria in response to mitochondrial depolarization.

### RalA and RalB negatively regulate TBK1 activity

Given that RalA and RalB both localize to mitochondria following mitochondrial depolarization, we sought to understand Ral’s function in these conditions. Previous studies found that Tank-binding kinase 1 (TBK1), a non-canonical IκB kinase, is recruited to depolarized mitochondria where it phosphorylates autophagy receptors to promote mitophagy [40,41]. Given that both RalA and RalB are implicated in TBK1 signaling [13–15] and that TBK1 exerts signaling effects in response to mitochondrial depolarization, we postulated that RalA and RalB may affect TBK1 activity in response to mitochondrial depolarization.

**Fig 7:**
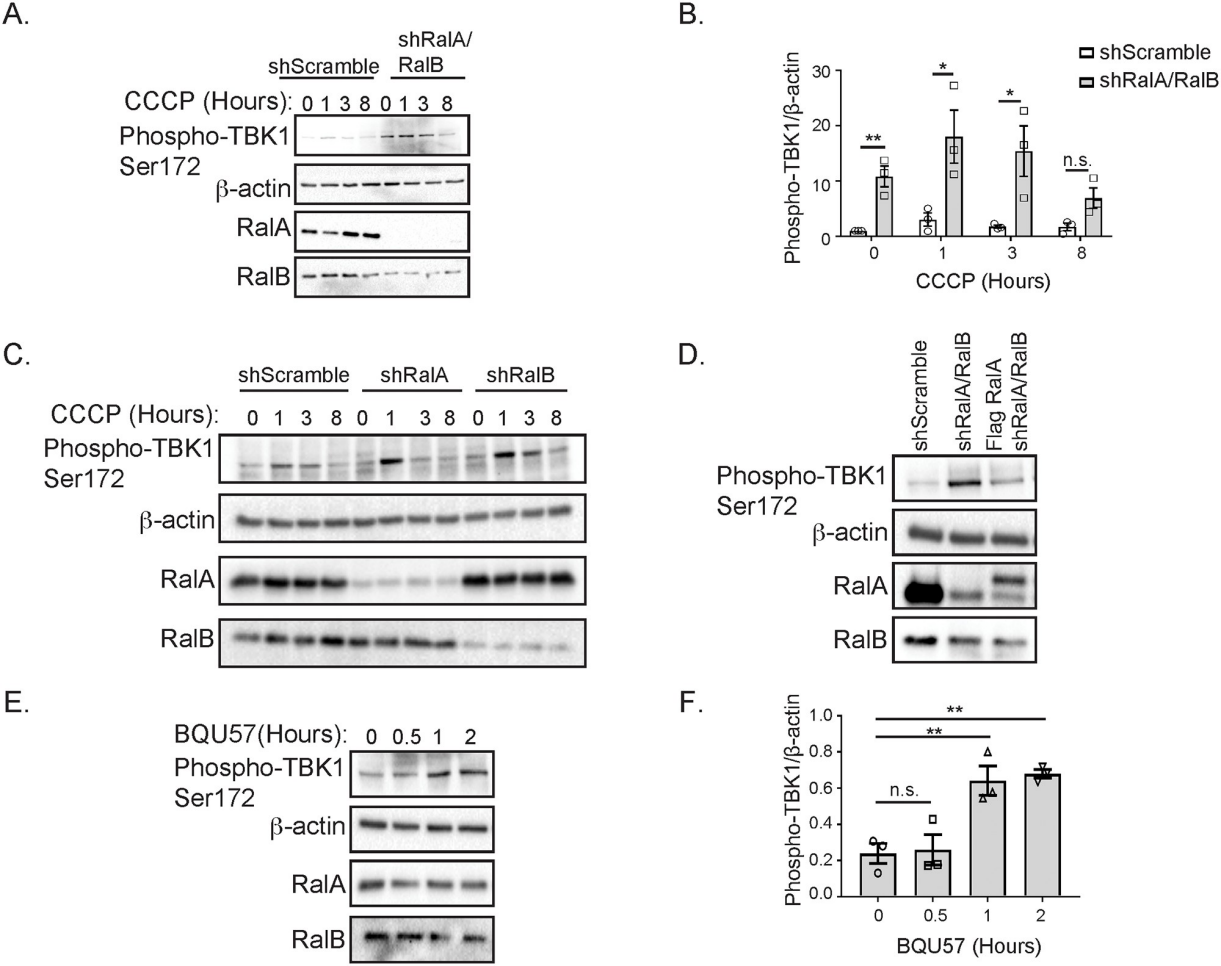
RalA and RalB negatively regulate TBK1 activity. (A) shScramble and shRalA/RalB HeLa cells were treated for 8 hours with 10 μM CCCP. An Immunoblot was performed for the indicated proteins. (B) Phospho-TBK1 levels in A were quantified with ImageJ, normalized to β-actin and then further normalized to the untreated value obtained for shScramble cells (Two-tailed Unpaired t-test, mean ± SEM, *p ≤ 0.05, **p ≤ 0.01, n.s. = not significant, n = 3). (C) Stably expressing shScramble, shRalA and shRalA HeLa cells were treated with 10 μM CCCP for 8 hours and immunoblotted for the indicated proteins. (D) HeLa cells stably expressing shScramble, shRalA/RalB or Flag RalA and shRalA/RalB constructs were immunoblotted for the indicated proteins. (E) HeLa cells were treated with 50 μM BQU57 for 2 hours and an Immunoblot was performed for the indicated proteins. (F) Phospho-TBK1 levels in E were quantified with ImageJ and normalized to β-actin (One-way ANOVA using Dunnett’s multiple comparisons test, mean ± SEM, **p ≤ 0.01, n.s. = not significant, n = 3).

We studied TBK1 activity in response to CCCP treatment in HeLa cells stably expressing shRNA targeting a scramble control sequence or RalA and RalB. As expected, following 1 hour of CCCP treatment shScramble HeLa cells exhibit increased TBK1 activity, which decreases over 8 hours (Fig 7A). In contrast to shScramble cells, shRalA/RalB double knockdown cells exhibit increased TBK1 activity both basally and in response to CCCP (Fig 7A and 7B). This phenotype is also observed, through to a lesser extent in single shRalA or shRalB HeLa cells (Fig 7C) indicating that RalA and RalB act redundantly in regulating TBK1 activity. These data suggest that RalA and RalB negatively regulate TBK1 activity.

To validate our finding that shRalA/RalB HeLa cells exhibit increased TBK1 activity both basally and following mitochondrial depolarization, we performed a rescue experiment by stably expressing Flag RalA in shRalA/RalB double knockdown cells (Fig 7D). Consistent with the idea that RalA and RalB act redundantly in negatively regulating TBK1 activity, we find that re-expression of Flag RalA is sufficient to reduce TBK1 activity in shRalA/RalB cells (Fig 7D).

To further confirm that Ral negatively regulates TBK1 activity, we treated HeLa cells over 2 hours with BQU57, a Ral inhibitor (Fig 7E). BQU57 inhibits both RalA and RalB by locking them in a GDP-bound state. We anticipated that inhibiting Ral would phenocopy the elevated phospho-TBK1 levels observed in shRalA/RalB cells (Fig 7A and 7B) because RalA-GTP levels decrease following mitochondrial depolarization (Fig 2A and 2B) and TBK1 activity rises in response to depolarization (Fig 7A). Consistent with this, TBK1 activity increases over 2 hours of BQU57 treatment (Fig 7E and 7F). Collectively, this data suggests that RalA and RalB negatively regulate TBK1 activity.

We next sought to determine if TBK1 activity affects RalA and RalB relocalization.

**Fig 8:**
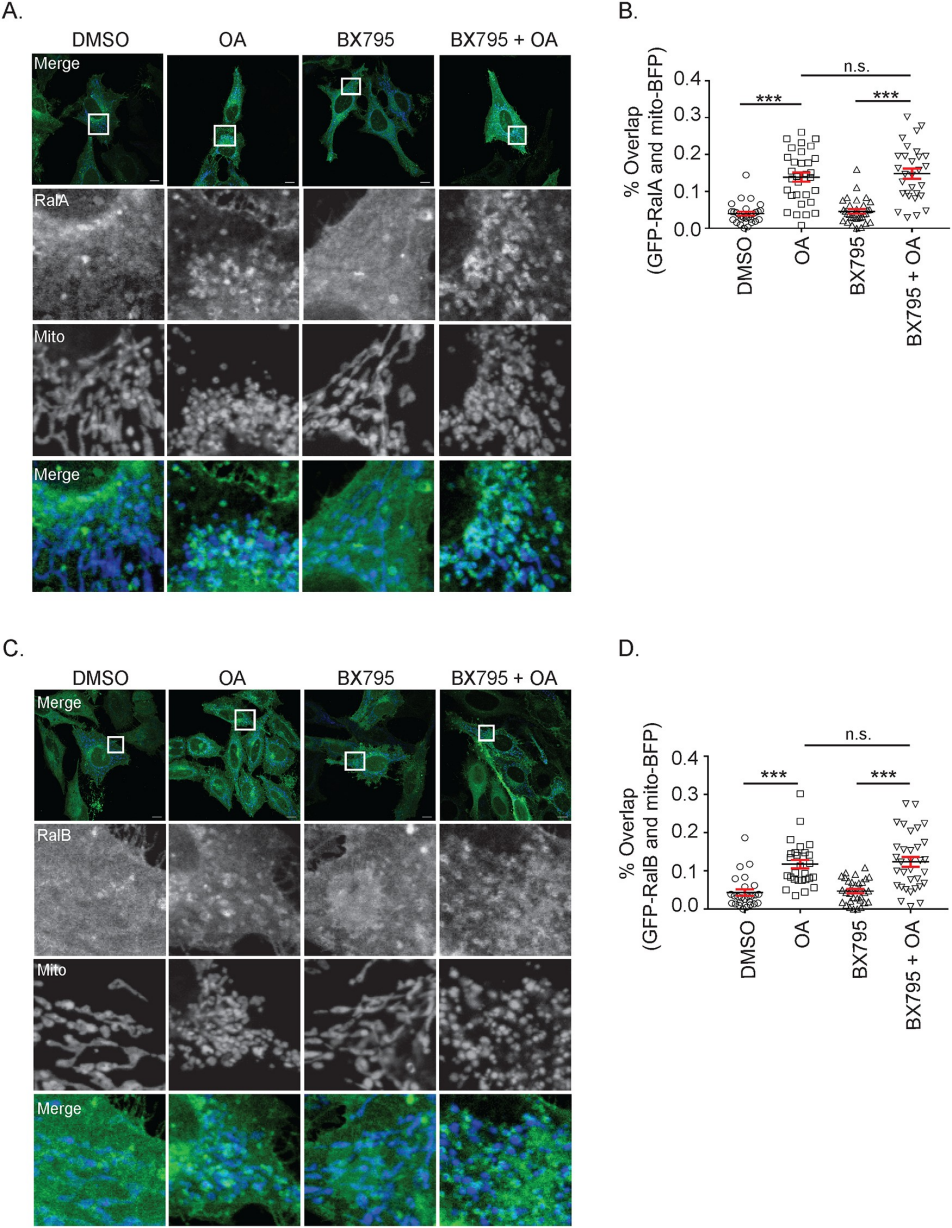
RalA and RalB relocalization to depolarized mitochondria occurs upstream of TBK1 activation. (A) HeLa cells transiently expressing GFP-RalA and mito-BFP were pre-treated with vehicle control (DMSO) or 2 μM BX795 for 1 hour and then treated for an additional hour as indicated. Cells were imaged by confocal microscopy (Scale bars = 10 μM). (B) Quantification of A to determine the degree of RalA colocalization with mitochondria (One-way ANOVA using Tukey’s multiple comparisons test, mean ± SEM, ***p ≤ 0.001, n.s. = not significant, n = 3). (C) Same experiment as in A except HeLa cells express GFP-RalB instead of GFP-RalA. (D) Quantification of C to determine the degree of RalB colocalization with mitochondria (One-way ANOVA using Tukey’s multiple comparisons test, mean ± SEM, ***p ≤ 0.001, n.s. = not significant, n = 3).

To address this, we induced mitochondrial depolarization in HeLa cells transiently expressing GFP-RalA or GFP-RalB and mito-BFP with OA in the presence or absence of BX795, a TBK1 inhibitor (Fig 8A and 8C). Both GFP-RalA and GFP-RalB relocalized to depolarized mitochondria in the presence of BX795, suggesting that Ral relocalization is independent of TBK1 activity (Fig 8A, 8B, 8C and 8D).

**Fig 9:**
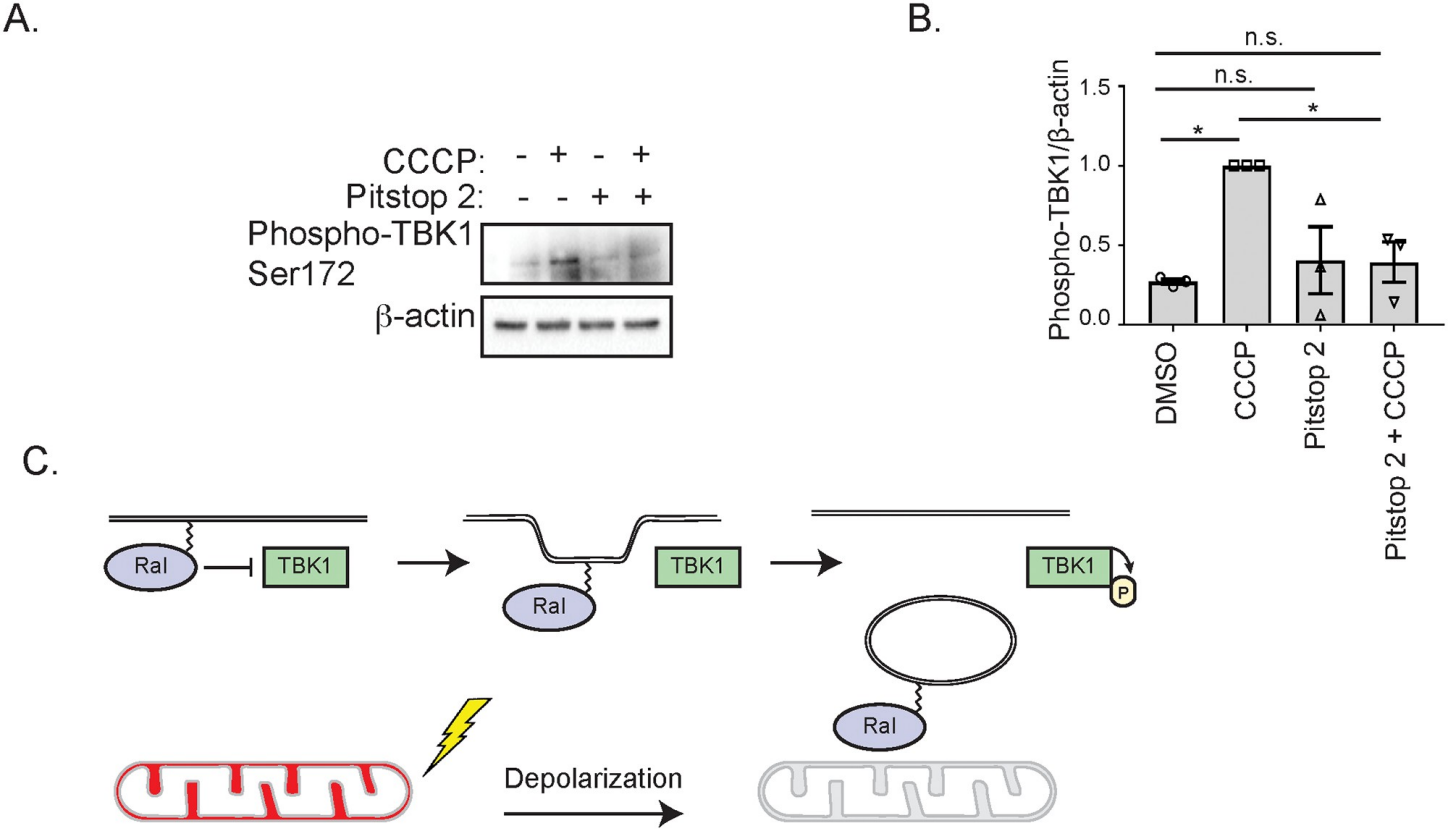
Ral relocalization affects TBK1 activity. (A) HeLa cells were pre-treated either with vehicle control (DMSO) or 20 μM Pitstop 2 for 15 minutes in serum-free DMEM containing 10 mM HEPES. Cells were then treated for 1 hour as indicated and immunoblotted for the indicated proteins. (B) Phospho-TBK1 levels in A were quantified with ImageJ, normalized to β-actin and then further normalized to the value obtained for cells treated 1 hour with CCCP treatment alone (One-way ANOVA using Tukey’s multiple comparisons test, mean ± SEM, *p ≤ 0.05, n.s. = not significant, n = 3). (C) Model of how RalA and RalB negatively regulate TBK1. Following mitochondrial depolarization, RalA and RalB localize to mitochondria via CME leading to a release of their inhibition of TBK1.

We next hypothesized that Ral relocalization following depolarization may affect its ability to inhibit TBK1 activity. To test this, we depolarized mitochondria in HeLa cells with CCCP in the absence and presence of Pitstop 2 and measured TBK1 phosphorylation. Consistent with a model in which Ral relocalization releases it from inhibiting TBK1, cells treated with both Pitstop 2 and CCCP, which fail to relocalize Ral to mitochondria (Fig 4B, 4C, 4D and 4E) also fail to exhibit increased TBK1 activity (Fig 9A and 9B). Importantly, treatment with Pitstop 2 alone does not affect TBK1 activity (Fig 9A and 9B). Collectively, these data support a model where RalA and RalB inhibit TBK1 under basal conditions and their relocalization following mitochondrial depolarization releases the inhibition, allowing TBK1 activity to increase (Fig 9C).

## Discussion

Mitochondrial depolarization initiates a number of different cellular processes, including changes in mitochondrial dynamics and mitophagy. Here we show that both RalA and RalB relocalize to depolarized mitochondria (Fig 1A, 1C, 1D and 1F). We previously demonstrated that RalA localizes to mitochondria during mitosis [8] and our data here suggests that the role of Ral proteins in regulating mitochondrial structure and function are more widespread. We find that RalA-GTP levels decrease in response to mitochondrial depolarization. However, neither RalA nucleotide-binding nor phosphorylation on serine 183 or serine 194 are required for RalA redistribution to depolarized mitochondria. In contrast, we find that CME facilitates Ral localization following mitochondrial depolarization. It remains to be determined whether Ral directly associates with endosomes and how endocytic trafficking enables delivery of Ral to mitochondrial membranes. It is possible that RalA and RalB associate with the endosomal pathway through direct protein-protein interactions. Additionally, post-translational modifications other than the ones tested in this paper may contribute to Ral’s trafficking and localization. For instance, both RalA and RalB undergo nondegradative ubiquitination that has previously been shown to regulate localization [42]. Specifically, de-ubiquitination of RalA promotes its relocalization from the plasma membrane to lipid raft microdomains.

Surprisingly, we find that RalA and RalB inhibit TBK1, both basally and in response to mitochondrial depolarization. Previous studies have shown that Ral GTPases promote TBK1 activity and are required for TBK1 function [13,15]. Our data suggest the relationship between Ral and TBK1 is likely more complex and may depend on context-specific factors.

Interestingly, we observe that CME participates both in TBK1 activation and Ral relocalization in response to mitochondrial depolarization. As such, we propose a model in which Ral inhibition of TBK1 activity is location-dependent. In other words, redistribution of Ral is a mechanism through which its inhibition of TBK1 can be removed (Fig 9C). It is tempting to speculate that this mechanism also involves the disruption of a protein complex between Ral, Ral effectors and TBK1. This would be consistent with the decrease in Ral-GTP levels observed following depolarization and the previous reports demonstrating that Ral and TBK1 interact through the Ral effector, Sec5.

Our data raises the important question of how regulation of TBK1 activity contributes to the cellular response to mitochondrial depolarization, if at all. TBK1 is known to play roles in autophagy, through induction of p62 [43] and in the inflammatory response, through induction of IRF3 and NFκB. It is plausible that the constitutive inhibition of TBK1 by RalA and RalB allows these response pathways to be poised and ready to respond rapidly in the case that mitochondrial damage occurs.

With this in mind, it is also possible that mitochondrial depolarization triggers an inflammatory response. The release of mitochondrial DNA (mtDNA) is seen as foreign to the body and results in an innate inflammatory response [44]. A previous study demonstrated that mtDNA activates the signaling molecule STING, which then signals to TBK1 to promote type I interferon responses [45]. Ostensibly, sustained mitochondrial depolarization could result in the release of mtDNA, initiating a TBK1-mediated inflammatory response. Thus, it is possible that Ral inhibits TBK1 activity basally to hinder an inappropriate inflammatory response.

Although the functional consequences of Ral-mediated TBK1 inhibition remain to be determined, our data show that RalA and RalB localize to depolarized mitochondria in a CME-dependent process and inhibit TBK1 activity. We speculate that the redundant functions of RalA and RalB in inhibiting TBK1 activity underscore the importance of TBK1 regulation in preventing an inappropriate inflammatory or autophagic response. Finally, this work provides another example of Ral functioning at mitochondria and may provide additional hints into how mitochondrial depolarization may affect diseases such as cancers and neurodegenerative disorders.

## Supporting information

S1 Fig OA, Valinomycin and CCCP induce mitochondrial depolarization and RalA relocalization. (A) HeLa cells stably expressing mCherry-Parkin and transiently expressing mito-BFP and GFP-RalA were treated with 10 μM oligomycin and 4 μM antimycin a (OA) over 3 hours and imaged with confocal microscopy (Scale bars = 10 μM). (B) Same as A, except HeLa cells were treated with 10 μM valinomycin (Scale bars = 10 μM). (C) HeLa cells transiently expressing mCherry-Parkin and GFP-RalA were treated with 10 μM CCCP for 3 hours and imaged with confocal microscopy (Scale bars = 10 μM).
(TIFF)

S2 Fig GFP does not relocalize to depolarized mitochondria. (A) HeLa cells that transiently express mCherry-Parkin and either GFP or GFP-RalA were treated with 10 μM oligomycin and 4 μM antimycin a (OA) for 3 hours and imaged by confocal microscopy (Scale bars = 10 μM).
(TIFF)

S3 Fig Original uncropped blots. (A) Uncropped blots from Fig 2A. (B) Uncropped blots from Fig 2B. (C) Uncropped blots from Fig 2C. (D) Uncropped blots from Fig 5A. (E) Uncropped blots from Fig 7A. (F) Uncropped blots from Fig 7C. (G) Uncropped blots from Fig 7D. (H) Uncropped blots from Fig 7E. (I) Uncropped blots from Fig 9A.
(TIFF)
