# Supplementary figures and images for: RalA and RalB relocalization to depolarized mitochondria depends on clathrin-mediated endocytosis and facilitates TBK1 activation
Source: PLoS One. 2019 Apr 17;14(4):e0214764. doi: 10.1371/journal.pone.0214764 (PMC6469766; doi:10.1371/journal.pone.0214764)

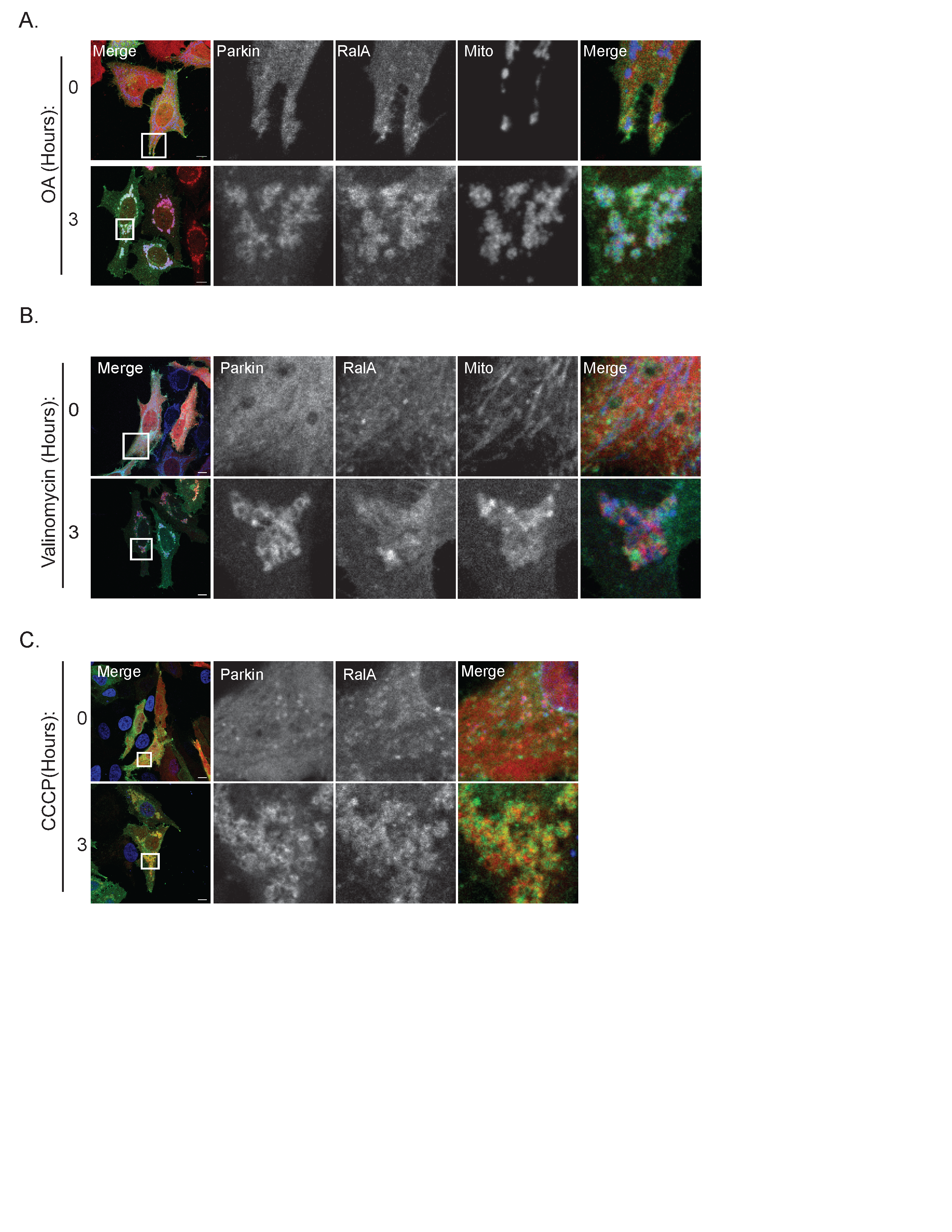

Supplement: S1 Fig — (A) HeLa cells stably expressing mCherry-Parkin and transiently expressing mito-BFP and GFP-RalA were treated with 10 μM oligomycin and 4 μM antimycin a (OA) over 3 hours and imaged with confocal microscopy (Scale bars = 10 μM). (B) Same as A, except HeLa cells were treated with 10 μM valinomycin (Scale bars = 10 μM). (C) HeLa cells transiently expressing mCherry-Parkin and GFP-RalA were treated with 10 μM CCCP for 3 hours and imaged with confocal microscopy (Scale bars = 10 μM). (TIFF) [file pone.0214764.s001.tiff]

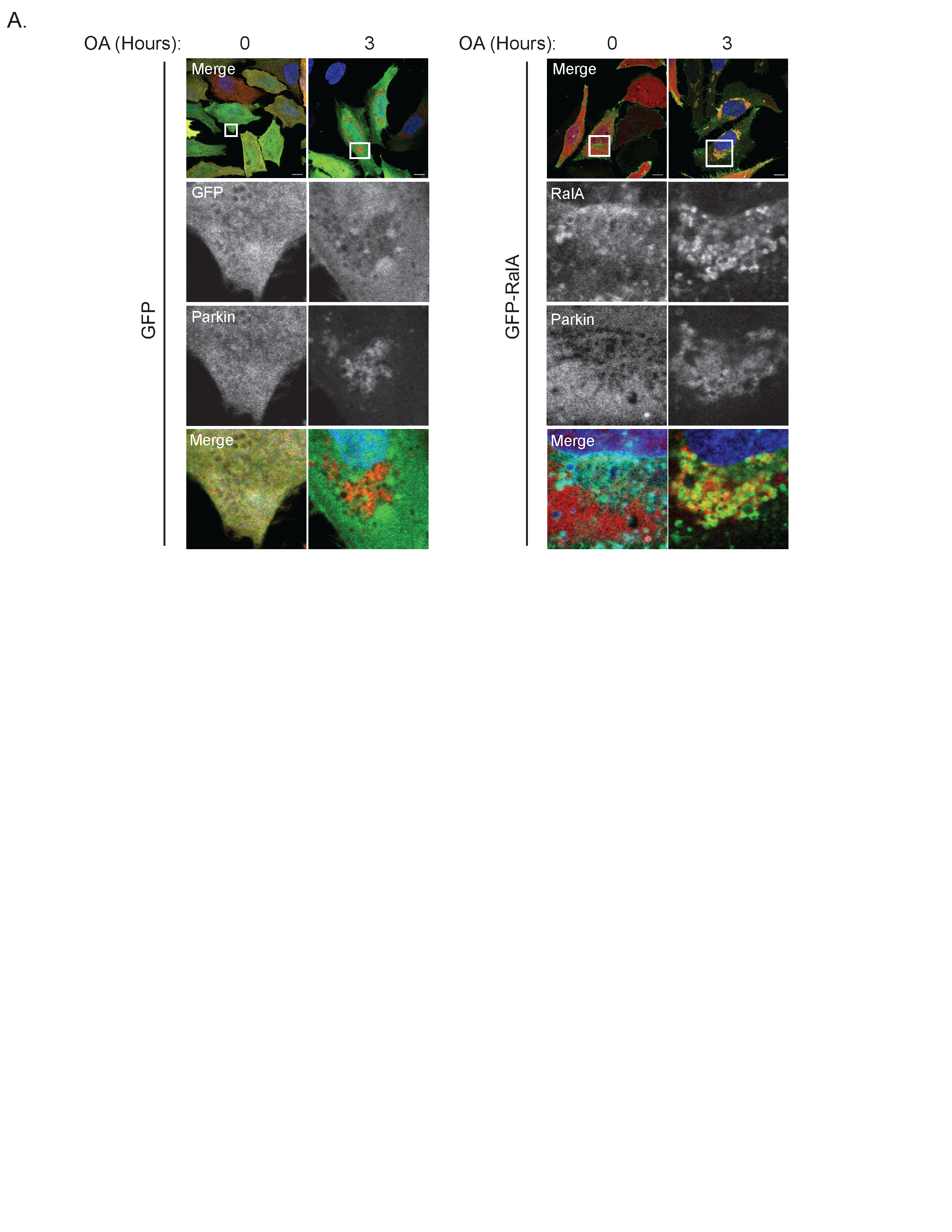

Supplement: S2 Fig — (A) HeLa cells that transiently express mCherry-Parkin and either GFP or GFP-RalA were treated with 10 μM oligomycin and 4 μM antimycin a (OA) for 3 hours and imaged by confocal microscopy (Scale bars = 10 μM). (TIFF) [file pone.0214764.s002.tiff]

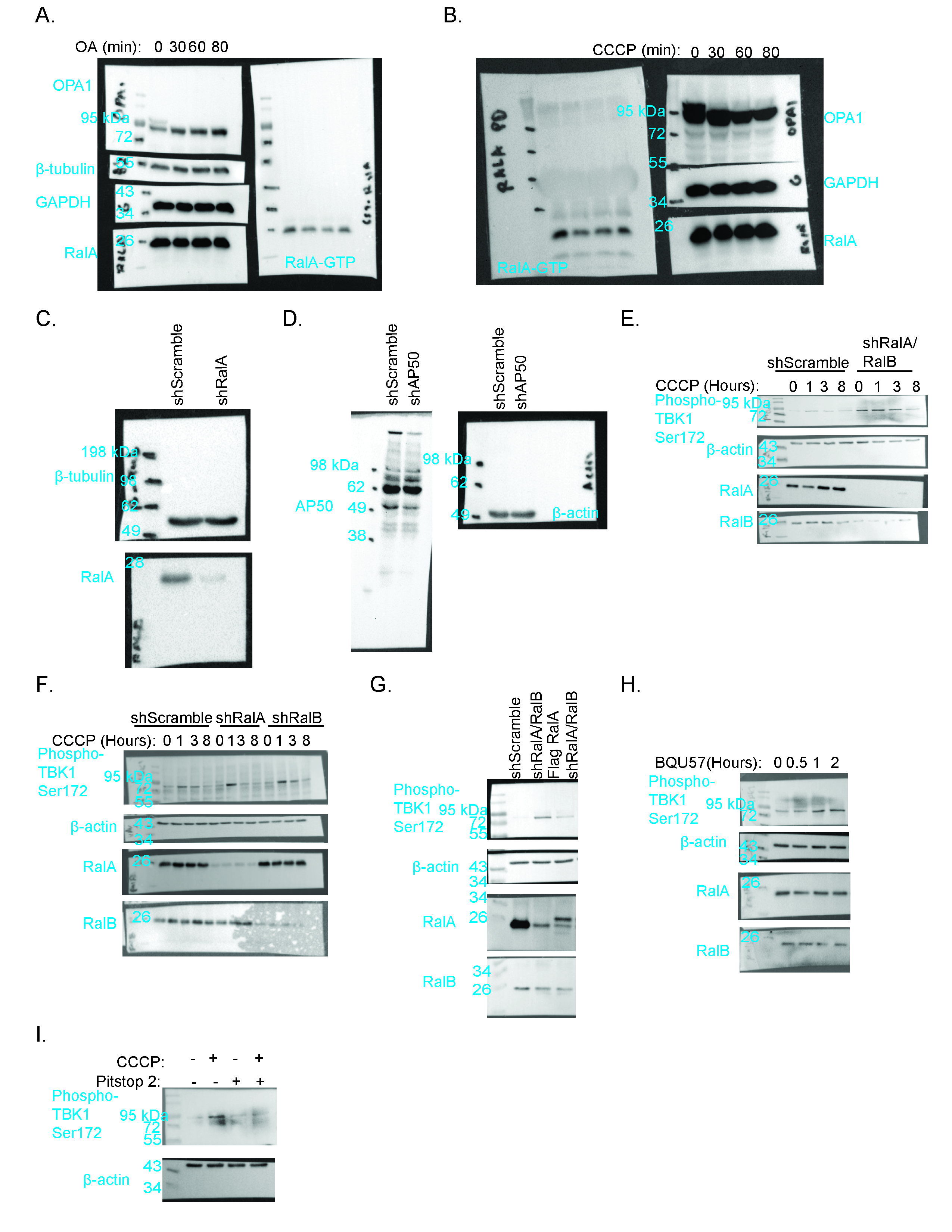

Supplement: S3 Fig — (A) Uncropped blots from Fig 2A. (B) Uncropped blots from Fig 2B. (C) Uncropped blots from Fig 2C. (D) Uncropped blots from Fig 5A. (E) Uncropped blots from Fig 7A. (F) Uncropped blots from Fig 7C. (G) Uncropped blots from Fig 7D. (H) Uncropped blots from Fig 7E. (I) Uncropped blots from Fig 9A. (TIFF) [file pone.0214764.s003.tiff]
